# Target of Rapamycin Regulates Genome Methylation Reprogramming to Control Plant Growth in *Arabidopsis*

**DOI:** 10.3389/fgene.2020.00186

**Published:** 2020-03-03

**Authors:** Tingting Zhu, Linxuan Li, Li Feng, Huijuan Mo, Maozhi Ren

**Affiliations:** ^1^Institute of Urban Agriculture, Chinese Academy of Agricultural Sciences, Chengdu, China; ^2^School of Life Sciences, Chongqing University, Chongqing, China; ^3^Zhengzhou Research Base, State Key Laboratory of Cotton Biology, Zhengzhou University, Zhengzhou, China

**Keywords:** target of rapamycin, DNA methylation, AZD-8055, rapamycin, plant growth, *Arabidopsis*

## Abstract

DNA methylation is an indispensable epigenetic modification that dynamically regulates gene expression and genome stability during cell growth and development processes. The target of rapamycin (TOR) has emerged as a central regulator to regulate many fundamental cellular metabolic processes from protein synthesis to autophagy in all eukaryotic species. However, little is known about the functions of TOR in DNA methylation. In this study, the synergistic growth inhibition of *Arabidopsis* seedlings can be observed when DNA methylation inhibitor azacitidine was combined with TOR inhibitors. Global DNA methylation level was evaluated using whole-genome bisulfite sequencing (WGBS) under TOR inhibition. Hypomethylation level of whole genome DNA was observed in AZD-8055 (AZD), rapamycin (RAP) and AZD + RAP treated *Arabidopsis* seedlings. Based on functional annotation and KEGG pathway analysis of differentially methylated genes (DMGs), most of DMGs were enriched in carbon metabolism, biosynthesis of amino acids and other metabolic processes. Importantly, the suppression of TOR caused the change in DNA methylation of the genes associated with plant hormone signal transduction, indicating that TOR played an important role in modulating phytohormone signals in *Arabidopsis*. These observations are expected to shed light on the novel functions of TOR in DNA methylation and provide some new insights into how TOR regulates genome DNA methylation to control plant growth.

## Introduction

DNA methylation is an important part of epigenetics, which is widely distributed in microbes, animals and plants. DNA methylation plays an important role in controlling transcriptional silencing of transposon, regulating gene expression and maintaining plant development (Moore et al., 2013; Bouyer et al., 2017; Zhang et al., 2018), which is one of the most studied epigenetic modifications in epigenetics. The methyl of DNA methylation provided by *S*-adenosylmethionine is transferred to the cytosine of genome DNA under the catalysis of DNA methyltransferase (Razin and Riggs, 1980). Mammals mainly methylate cytosine at symmetrical CG site, while plant DNA methylation occurs in all cytosine sequence contexts: CG, CHG, and CHH (H represents A, T, or C) (Lister et al., 2008). DNA methylation regions are mainly found in highly repetitive sequences (transposon and rDNA), promoter region (suppressing gene expression), coding sequence region and intergenic region. More than 5% of the expressed genes have DNA methylation in their promoter region, and more than 33% of genes contain DNA methylation within the coding sequence region in *Arabidopsis* (Zhang et al., 2006). Promoter-methylated genes are low expressed and show a greater degree of tissue specific expression, whereas genes methylated in transcribed regions are highly expressed (Zhang et al., 2006). However, recently study also showed that methylation in transcribed regions can negatively regulate the gene expression (Long et al., 2014; Lou et al., 2014).

DNA methylation is critically important for normal growth and development in both animals and plants; null mutations of DNA methyltransferase DNMT1 or DNMT3 result in embryonic lethality in mouse, and *drm1*/*drm2*/*cmt3* triple mutants exhibit developmental abnormalities in *Arabidopsis* (Grace and Bestor, 2005; Chan et al., 2006). 5-Azacytidine (Azacitidine) is a nucleoside analog of cytidine that specifically inhibits DNA methylation by capturing DNA methyltransferase in bacteria and mammalian (Christman, 2002). In plants, genome-wide demethylation caused by methylation inhibitor azacitidine leads to growth retardation, malformations, and changes in the flowering time or flower sexuality (Fieldes et al., 2005; Marfil et al., 2012). Interestingly, azacitidine can increase amounts of somatic embryos in somatic embryogenesis stage, indicating that DNA demethylation caused by azacitidine promotes the reprogramming of gene expression, acquisition of totipotency and initiation of embryogenesis in explant (Osorio-Montalvo et al., 2018).

The target of rapamycin (TOR) is an evolutionarily conserved protein kinase that integrates nutrient and energy signaling to regulate growth and homeostasis in fungi, animals and plants. TOR is activated by both nitrogen and carbon metabolites and promotes energy-consuming processes such as mRNA translation, protein biosynthesis and anabolism while represses autophagy and catabolism in times of energy abundance (Dobrenel et al., 2016; Juppner et al., 2018; Ahmad et al., 2019). However, deregulated mammalian target of rapamycin (mTOR) signaling is implicated in the progression of cancer and diabetes, and the aging process in mammalian (Saxton and Sabatini, 2017). Genetic, physiological and genomic studies revealed that TOR plays central roles in plant embryogenesis, seedling growth, root and shoot meristem activation, root hair elongation, leaf expansion, flowering and senescence (Ren et al., 2011, 2012; Xiong et al., 2013; Yuan et al., 2013; Xiong and Sheen, 2014; Deng et al., 2017; Shi et al., 2018). *TOR* gene was originally identified by genetic mutant screens for resistance to rapamycin in budding yeast (Heitman et al., 1991). Subsequent research showed that null mutation of *tor* resulted in embryonic lethality in yeast, animals and plants (Heitman et al., 1991; Ren et al., 2011; Saxton and Sabatini, 2017), indicating that TOR was an essential kinase in eukaryotes. Since rapamycin acts as a specific inhibitor of the TOR kinase, the TOR signaling pathway is quickly considered as a central regulator by application of rapamycin in yeast and animals (Benjamin et al., 2011). However, TOR is insensitive to rapamycin in plants, which is mainly due to evolutionary mutation of the *FK506*-*binding protein 12* (*FKBP12*) gene, resulting in loss of function to bind rapamycin (Xu et al., 1998). To dissect TOR signaling pathway in *Arabidopsis* by using rapamycin, Ren et al. (2012) generated a rapamycin-hypersensitive line (BP12-2) by introducing yeast *FKBP12* gene into *Arabidopsis*. Inhibition of AtTOR in BP12-2 line by rapamycin resulted in slower root, shoot and leaf growth and development, leading to poor carbon and nitrogen metabolism, nutrient uptake and light energy utilization (Ren et al., 2012). Additionally, the ATP competitive TOR kinase inhibitors including Torin2, WYE-132, Ku-0063794, and AZD-8055 (AZD) were also applied to study the TOR pathway in plants (Montane and Menand, 2013; Li et al., 2015; Song et al., 2017, 2018). As revealed by recent studies, AZD had high specificity and strong inhibitory effects on TOR activity in flowering plants (Montane and Menand, 2013; Li et al., 2015), implying that AZD can be preferentially applied to plants to dissect TOR signaling pathway compared with other TOR kinase inhibitors in angiosperms.

The TOR signaling pathway is a central regulator in regulating cell growth, homeostasis, proliferation and metabolism (Dobrenel et al., 2016; Saxton and Sabatini, 2017; Shi et al., 2018). DNA methylation is an epigenetic mechanism that plays key roles in genome integrity, suppression of transposon, gene expression and somatic embryogenesis in plants (Osorio-Montalvo et al., 2018; Zhang et al., 2018). However, it has not been reported whether TOR directly or indirectly regulates the methylation level of genome DNA to control plant growth and development. In this study, we performed base-resolution whole-genome bisulfite sequencing (WGBS) under TOR inhibition in *Arabidopsis*. Differentially methylated regions and genes support the evolutionarily conserved TOR functions in ribosome biogenesis, metabolism, and cell growth. Our detailed genome-wide analysis of DNA methylation under TOR inhibition provides new insights into how TOR regulates global DNA methylation to control plant growth.

## Materials and Methods

### Plant Materials and Growth Conditions

WT *Arabidopsis* Columbia (Col-0) and the transgenic *Arabidopsis* BP12-2 line were used in this study (Ren et al., 2012). Sterile treatment of *Arabidopsis* seeds surface prior to plating. The seeds first repeatedly were shook in 75% ethanol for 2 min and the supernatant was discarded. Then, shaking the seeds repeatedly with 10% sodium hypochlorite containing 0.3% Tween-20 for 4 min, and discarding the supernatant; followed by four or five rinses with sterile water, and the supernatant was discarded. Finally, the seeds were suspended in 0.15% sterile agarose solution and kept at 4°C for 2 days. Sterilized seeds were plated on plates, and then grown in a controlled environment at 22°C under 16 h 60–80 μE⋅m^–2^ s^–1^ continuous light and 8 h darkness.

### DNA Library Construction and Whole-Genome Bisulfite Sequencing

BP12-2 seedlings of 7 days were treated with DMSO, AZD (1 μM), RAP (5 μM), and AZD (1 μM) + RAP (5 μM) for 24 h, and each sample contained three biological replicates. Total genomic DNA was extracted using a plant genomic DNA kit (TIANGEN, Beijing, China) according to the manufacturer’s instructions. Genomic DNA degradation and contamination was monitored on agarose gels. DNA purity was checked using the NanoPhotometer^®^ spectrophotometer (IMPLEN, Westlake Village, CA, United States). DNA concentration was measured using Qubit^®^ DNA Assay Kit in Qubit^®^ 2.0 Fluorometer (Life Technologies, CA, United States). A total amount of 5.2 microgram genomic DNA spiked with 26 ng lambda DNA were fragmented by sonication to 200–300 bp with Covaris S220, followed by end repair and adenylation. Cytosine-methylated barcodes were ligated to sonicated DNA as per manufacturer’s instructions. Then these DNA fragments were treated twice with bisulfite using EZ DNA Methylation-Gold Kit^TM^ (Zymo Research). In addition, the resulting single-strand DNA fragments were PCR amplificated using KAPA HiFi HotStart Uracil + ReadyMix (2X). Library concentration was quantified by Qubit^®^ 2.0 Fluorometer (Life Technologies, CA, United States) and quantitative PCR, and the insert size was checked on Agilent Bioanalyzer 2100 system. The clustering of the index-coded samples was performed on a cBot Cluster Generation System using TruSeq PE Cluster Kit v3-cBot-HS (Illumia) according to the manufacturer’s instructions. After cluster generation, the prepared library were sequenced on an Illumina Hiseq 2000/2500 platform, and 100/50 bp single-end reads were generated. Image analysis and base calling were performed with the standard Illumina pipeline, and finally 100 bp paired-end reads were generated.

### Estimating Methylation Level

To identify the methylation level, we employed a sliding-window approach, which was conceptually similar to approaches that have been used for bulk BS-Seq. With window size = 3,000 bp and step size = 600 bp (Smallwood et al., 2014), the sum of methylated cytosine (mC) and unmethylated cytosine (C) read counts in each window were calculated. Methylation level (ML) for each cytosine site showed the fraction of methylated C, and was defined as: ML (mC) = reads (mC)/reads (mC) + reads (C). Calculated ML was further corrected with the bisulfite non-conversion rate according to previous studies (Lister et al., 2013).

### Analysis of Methylation Levels in Genomic Functional Regions

Analysis of the average methylation level of the CG, CHG, and CHH sites in genomic functional regions including promoter (2 kb region upstream of the transcription start site), 5′UTR, exon, intron and 3′UTR regions. Divided each functional element region in the genome annotation into 20 bins, and counted the number of mC and C reads in each bin. For average plots, average values in 20 bins were calculated and plotted.

### Differentially Methylated Regions (DMRs) Analysis

Differentially methylated regions (DMRs) were identified using the Bsseq R package software, which used a sliding-window approach (reads coverage ≥5). The window was set to 1,000 bp and step length was 100 bp. The main steps of identification DMR were as follows: First set the sliding window and sliding step size, every 1000 bp as a window and 100 bp as the step size. Selected the DNA methylation level difference value >0.1 and the DNA methylation difference fold-change >2 between the treatment and the control sample, and the number of cytosine >10 as potential DMRs. Next, probabilities were calculated using a Fisher’s exact test. The regions with significant differences (*p* < 0.05) were considered as DMRs. Then, moved to the next window with the step size and repeated the above steps to obtain DMRs information of the whole genome. FDR (FDR < 0.05) was used to correct the p value of all DMRs.

### GO and KEGG Enrichment Analysis of DMR-Related Genes (DMGs)

Gene Ontology (GO) enrichment analysis of genes related to DMRs was implemented by the GOseq R package (Young et al., 2010), in which gene length bias was corrected. GO terms with corrected *P*-value less than 0.05 were considered significantly enriched by DMGs. Kyoto Encyclopedia of Genes and Genomes (KEGG) (Minoru et al., 2008) is a database resource for understanding high-level functions and utilities of the biological system, such as the cell, the organism and the ecosystem, from molecular-level information, especially large-scale molecular datasets generated by genome sequencing and other high-through put experimental technologies^1^. We used KOBAS software (Mao et al., 2005) to test the statistical enrichment of DMGs in KEGG pathways.

### Quantitative Real-Time PCR

Total RNA of transgenic *Arabidopsis* BP12-2 seedlings which treated for 24 h in mediums containing DMSO, AZD (1 μM), RAP (5 μM), and AZD (1 μM) + RAP (5 μM) was isolated using the RNAprep Pure Plant Kit (TIANGEN, Beijing, China). Total RNA was reverse transcribed into cDNA using the PrimeScript R RT reagent kit (Takara, Dalian, China). Relative transcript levels were assayed by the CFX96 real-time PCR system (BIO-RAD, United States). *AtACTIN2* was used as an internal control. Real-time PCR primers were shown in Supplementary Table S1. Reaction was performed in a final volume of 20 μL containing 10 μL of 2 × Power Top Green qPCR SuperMix (TRANSGEN, Beijing, China). RNA relative quantification analyses were performed with the Bio-Rad CFX manager software. The data represented the mean ± SD of three independent experiments.

### Combination Index (CI) Value Measurement

Combination index (CI) values were used to evaluate the interaction between azacitidine and AZD/RAP. The degree of reagents interaction was based on synergistic effect (CI < 1), additive effect (CI = 1), or antagonism (CI > 1) (Chou, 2006). WT and BP12-2 seeds were sown on plates containing DMSO, azacitidine, RAP, AZD, and pairwise combination for 10 days, and then fresh weight was measured for CI value assessment. Experiments were repeated at least three times. The values of affected fraction (Fa) were calculated according to the CompuSyn software program (Chou and Talalay, 1984; Chou, 2006).

## Results

### Azacitidine and TOR Inhibitors Synergistically Inhibit Seedlings Growth in *Arabidopsis*

**FIGURE 1:**
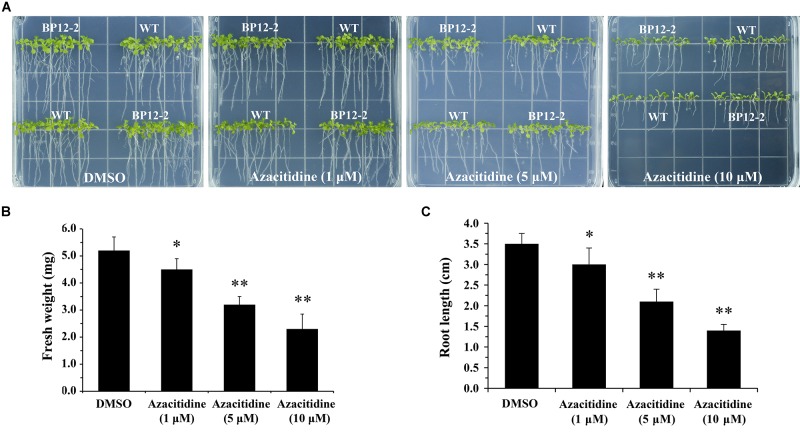
Azacitidine inhibits seedlings growth in dose-dependent manner in *Arabidopsis*. **(A)** Phenotypes of WT and BP12-2 seeds cultured on 1/2 MS medium containing increasing concentrations of azacitidine for 10 days. **(B,C)** Fresh weight and root length of WT seedlings growing on different azacitidine concentrations for 10 days. Each graph represents the average of 30 seedlings. Error bars indicate means ± SD of three biological replicates. Asterisks denote Student’s *t*-test significant difference compared with DMSO (^∗^*P* < 0.05, ^∗∗^*P* < 0.01).

Azacitidine is a specific inhibitor of DNA methylation, which interacts with DNA methyltransferase to inhibit DNA methylation in mammalian (Christman, 2002). To test the effect of azacitidine on seeds germination and seedlings growth in *Arabidopsis*, we treated *Arabidopsis* seeds with different concentrations of azacitidine. With the increase of azacitidine concentrations, Col-0 (WT) and BP12-2 seeds germination was not affected by azacitidine, whereas the seedlings growth was subjected to different degrees of inhibition, reflecting in the reduction of fresh weight and shorter root length (Figure 1). The 50% growth inhibitory dose (GI50) of azacitidine was 10 μM in accordance with fresh weight (Figure 1B). The phenotype of azacitidine-treated *Arabidopsis* seedlings is similar to that of TOR kinase inhibitors, implying that TOR may play a role in regulating DNA methylation in *Arabidopsis*. Interestingly, the transcription level of *AtTOR* did not significantly change in azacitidine-treated WT and BP12-2 seedlings (Supplementary Figure S1A), indicating that azacitidine had no effect on TOR expression.

**FIGURE 2:**
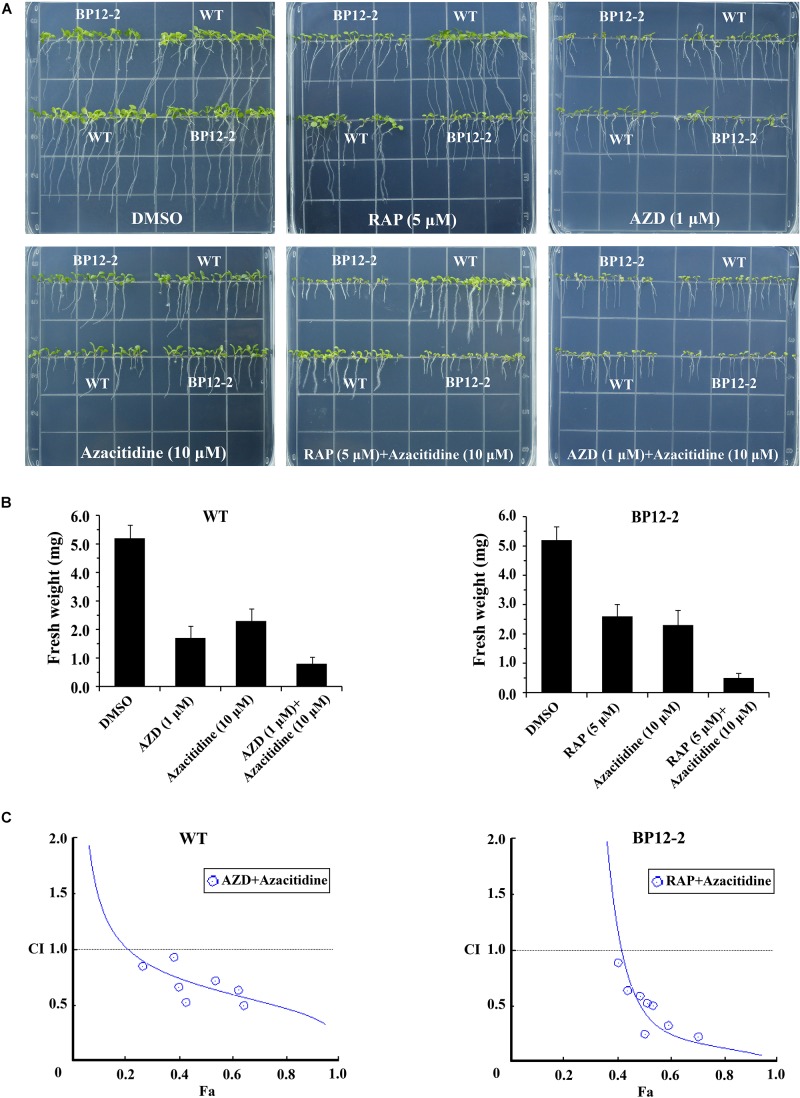
Azacitidine and TOR inhibitors synergistically inhibit seedlings growth in *Arabidopsis*. **(A)** Phenotypes of 10-day-old WT and BP12-2 seeds sown on 1/2 MS medium containing DMSO, azacitidine (10 μM), RAP (5 μM), AZD (1 μM), and the combination of RAP (5 μM) + azacitidine (10 μM) and AZD (1 μM) + azacitidine (10 μM). **(B)** Fresh weight of WT and BP12-2 seedlings sown on different plates for 10 days. Each graph represents the average of 30 seedlings. Error bars indicate means ± SD of three biological replicates. **(C)** Azacitidine and TOR inhibitors synergistically inhibit plant growth *in vitro*. WT and BP12-2 seeds were sown on plates containing DMSO, azacitidine, RAP, AZD, and pairwise combination for 10 days, and then fresh weight was measured for CI value assessment. The Fa-CI curve shows synergistic effects (CI < 1) between AZD + azacitidine and RAP + azacitidine in WT and BP12-2 seedlings, respectively.

To examine the roles of TOR in the regulation of DNA methylation, we used combinations of TOR inhibitors and azacitidine to treat *Arabidopsis* seeds. Rapamycin (RAP) and AZD-8055 (AZD) that act as different types of TOR kinase inhibitors were selected to treat WT and BP12-2 *Arabidopsis* seeds. Consistent with the previous reports (Ren et al., 2012), RAP had no obvious inhibitory effect on WT seedlings, whereas significantly inhibited roots and shoots elongation and leaves expansion in BP12-2 seedlings (Figure 2A). The combination of RAP and azacitidine enhanced the inhibition of seedlings growth compared with RAP or azacitidine alone treatment, resulting in leaves yellowing and growth retardation in BP12-2 seedlings (Figures 2A,B). Meanwhile, the combination of AZD and azacitidine also enhanced the inhibition of seedlings growth, implying that TOR inhibitors and azacitidine may synergistically inhibit seedlings growth in *Arabidopsis*. To further explore whether TOR inhibitors and azacitidine synergistically inhibit seedlings growth, we used a combination index (CI) to calculate the interaction between TOR inhibitors and azacitidine in *Arabidopsis*. The combination treatment of RAP and azacitidine generated a strong synergistic effect (CI < 0.5) in BP12-2 seedlings. Meanwhile, the combination treatment of AZD and azacitidine also generated the synergistic effects (CI < 1) in WT plants (Figure 2C). These results indicated that TOR inhibitors and azacitidine synergistically inhibit the growth of *Arabidopsis* seedlings, implying the functions of TOR in DNA methylation.

### Inhibition of TOR Reduces Whole-Genome Methylation Level in *Arabidopsis*

**FIGURE 3:**
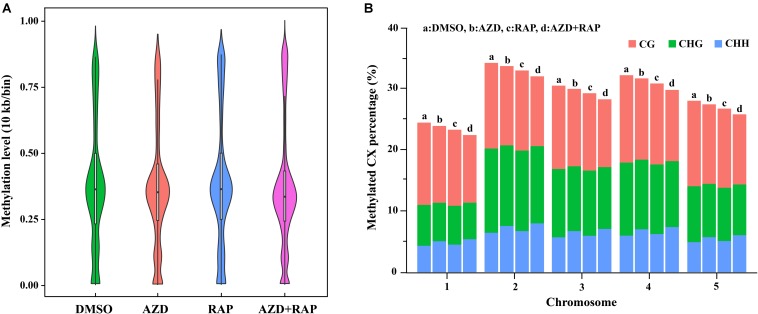
Genome-wide methylation level and distribution of mCG, mCHG and mCHH on chromosomes. **(A)** Methylation level distribution of whole genome in different samples. Take 10 Kb as a bin. The width of each violin represents the number of points at the methylation level. **(B)** The distribution of mCG, mCHG, and mCHH in all methylated cytosine on chromosomes. The *X*-axis shows chromosomes, the *Y*-axis represents the proportion of the methylation level on the corresponding chromosomes, different colors represent different context. a: DMSO, b: AZD, c: RAP, d: AZD + RAP.

To further analyze the functions of TOR in the regulation of DNA methylation, we performed base-resolution whole-genome bisulfite sequencing (WGBS) under TOR inhibition by AZD, RAP, and AZD + RAP treatment in *Arabidopsis*. Each sample contained more than 51 million clean reads after removing the low-quality reads, duplicate reads and adapters. The bisulfite conversion efficiency exceeded 99.5% in all samples, providing a reliable guarantee of the accuracy of WGBS (Table 1). We used Bowtie2 (Bismark) software to map the clean reads to the reference genome, and more than 58% of the reads were uniquely mapped to the *Arabidopsis* genome in each sample (Table 1). Further statistical analysis found that DNA methylation occurred mainly at three different sequence sites: CG, CHG, and CHH sites (H = A, T, or C) in all samples, we calculated methylation ratio of the three sequence contexts in the genome. The methylation ratio of the CG sequence was the highest, followed by the CHG sequence and the CHH sequence in all samples (Table 2). Among them, the methylation ratio of CG context was decreased, while the methylation ratio of CHH context was increased under TOR inhibition. Importantly, the total mCX methylation ratio was reduced in TOR-inhibited samples compared to DMSO control group (Table 2). Furthermore, genome-wide methylation level was decreased in TOR-inhibited samples, of which the methylation level was most obviously decreased in AZD + RAP treated sample (Figure 3A). Additionally, we analyzed the proportion of methylated C site on each chromosome and found that the methylation ratio of CG site on each chromosome was higher than the CHG and CHH sites. Consistent with the above findings, inhibition of TOR also reduced the proportion of methylated CX sites on each chromosome (Figure 3B).

**TABLE 1:** Data generated by whole-genome bisulfite sequencing (WGBS).

**Samples**	**Raw reads**	**Clean reads**	**GC content**	**Total reads**	**Mapped reads**	**Mapping rate**	**Uniquely mapping rate**	**Bisulfite conversion rate**
DMSO	56035294	53295965	20.12%	26438527	19114111	71.97%	58.56%	99.56%
AZD	59270545	55453129	20.43%	27726565	19734425	71.22%	59.59%	99.62%
RAP	57042080	51115347	20.35%	25557673	18379669	71.20%	59.48%	99.59%
AZD + RAP	64675954	58197908	20.45%	29098954	20895769	71.85%	58.49%	99.56%

**TABLE 2:** The proportion of methylated C site in the genome.

**Samples**	**mCpG (%)**	**mCHG (%)**	**mCHH (%)**	**Total mCX (%)**
DMSO	29.17	15.41	5.29	49.87
AZD	27.66	15.68	6.23	49.57
RAP	27.96	15.18	5.53	48.67
AZD C RAP	27.01	15.24	6.58	48.83

**FIGURE 4:**
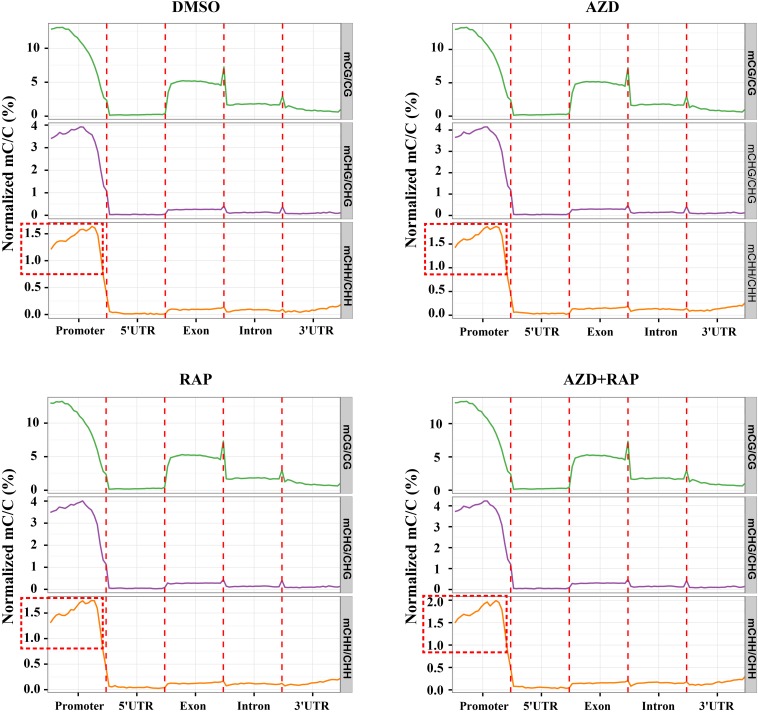
Distribution of methylation levels of all samples on different genomic elements. Abscissa represented different genomic elements, ordinate represented the average level of methylation, and different colors represented different sequence contexts (CG, CHG, and CHH). The promoter region is a 2 kb region upstream of the TSS site.

To explore the role of DNA methylation in regulating gene expression, we analyzed the changes of DNA methylation levels on genomic functional elements including promoters, exons, introns and UTR regions. Similar methylation levels were observed in the three methylated CX contexts in each functional element of all samples. Among them, the promoter had the highest DNA methylation ratio, followed by the exon region, and the 5′UTR had the lowest DNA methylation ratio in all samples (Figure 4). Interestingly, we found that inhibition of TOR increased the average methylation level of mCHH in the promoter region whereas mCG and mCHG had no obvious change, implying that TOR plays an important role in regulation of methylation of CHH site in promoter region. To investigate whether the reduction in genome-wide methylation was caused by methyltransferases, we examined the transcription levels of methyltransferase and demethylase genes under TOR inhibition. The transcription level of *METHYLTRANSFERASE 1* (*MET1*) that maintains CG methylation in plants was down-regulated in TOR-inhibited seedlings (Supplementary Figure S2A). However, *DOMAINS REARRANGED METHYLASE 1* (*DRM1*) and *DRM2* genes, which maintain asymmetric CHH site methylation in plants (Chan et al., 2005), were up-regulated under TOR inhibition, which could account for high methylation level of CHH site in the promoter region under TOR inhibition. Besides, the transcription levels of demethylase genes including *ROS1*, *MBD7*, and *IBM1* were significantly up-regulated in TOR-inhibited seedlings (Supplementary Figure S2B). These results indicated that TOR regulated DNA methylation by altering the transcription levels of methyltransferase and demethylase genes in *Arabidopsis*.

### Analysis of Differentially Methylated Region (DMR) Under TOR Inhibition

**FIGURE 5:**
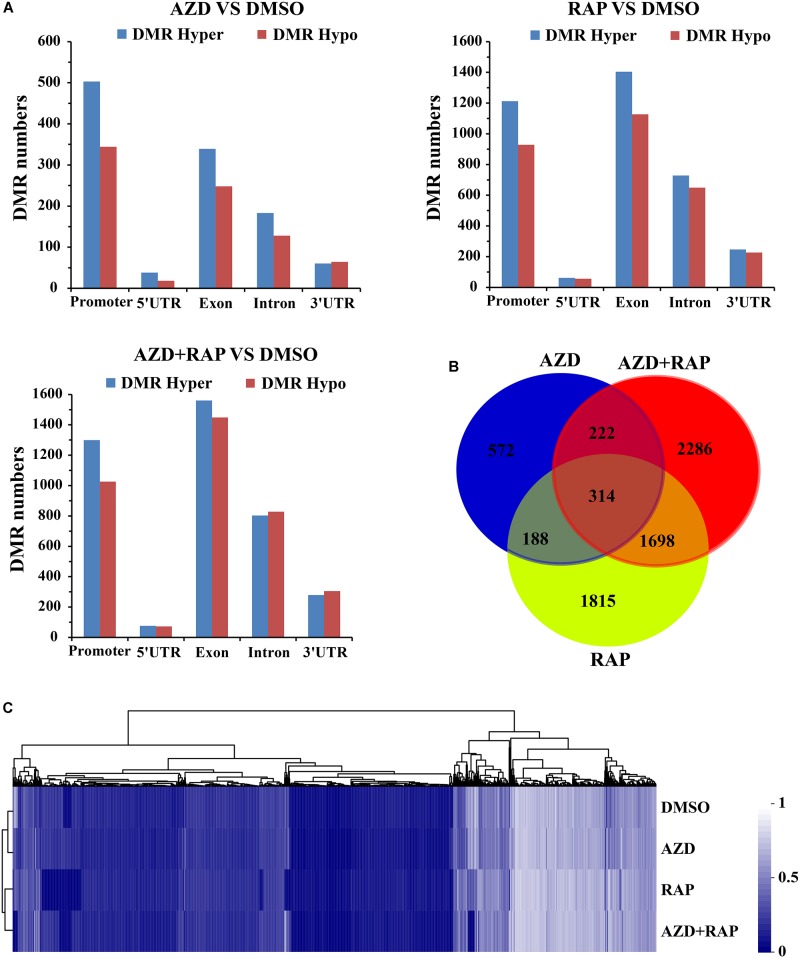
Differentially methylated regions (DMRs) analysis of DMSO, AZD, RAP, and AZD + RAP samples. **(A)** The numbers of DMRs in genome elements. Histograms showing the overall DMRs numbers of genome elements: promoter, 5′UTR, exon, intron, and 3′UTR regions. Hyper: high methylation level, hypo: low methylation level. **(B)** The Venn diagram of differentially methylated genes (DMGs) among different combinations of AZD vs. DMSO, RAP vs. DMSO, and AZD + RAP vs. DMSO groups. **(C)** Cluster analysis of DMGs for DMSO, AZD, RAP, and AZD + RAP treated samples. The blue color represented lower methylation level and the white color represented higher methylation level. Each row represented a sample, each column represented a gene.

Whole genome differential methylation analysis was performed in AZD vs. DMSO, RAP vs. DMSO, and AZD + RAP vs. DMSO groups. Total 1417, 4664, and 5282 DMRs were identified in AZD vs. DMSO, RAP vs. DMSO, and AZD + RAP vs. DMSO groups, respectively. All DMRs were classified into five types according to genome elements, most of which were distributed in promoter and exon regions. Moreover, hypermethylated DMRs were more than hypomethylated DMRs under TOR inhibition, of which hypermethylated DMRs were also mainly distributed in promoter and exon regions (Figure 5A). We further mapped the obtained DMRs of promoter, 5′UTR, exon, intron, and 3′UTR to genes. A total of 1296, 4015, and 4520 differentially methylated genes (DMGs) were found in AZD vs. DMSO, RAP vs. DMSO, and AZD + RAP vs. DMSO groups, respectively. The Venn diagram displayed that 314 DMGs were overlapping among three groups, while approximately 50% of the DMGs were not overlapping between these groups (Figure 5B). Furthermore, hierarchical cluster analysis of DMGs was performed using Cluster software, and methylated genes were clustered using a distance metric based on the Pearson correlation. The results showed that some DMGs had a hypomethylated status under TOR inhibition (Figure 5C). Especially, some significant hypomethylated genes were found in RAP-treated seedlings (Supplementary Table S2).

### Gene Ontology (GO) and KEGG Pathway Enrichment Analysis of DMGs

**FIGURE 6:**
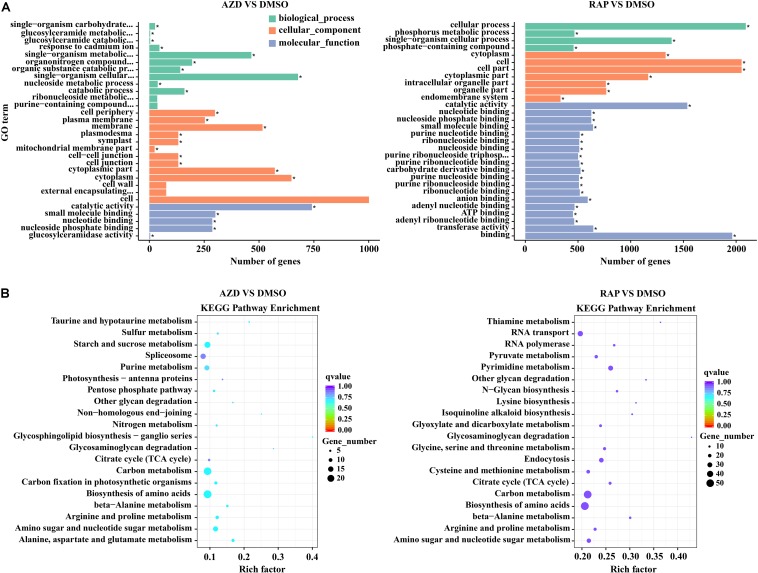
Gene ontology (GO) and KEGG pathway enrichment analysis of DMGs under TOR inhibition. **(A)** The top 30 most enriched GO terms analysis of DMGs. different colors represent biological processes, cellular components, and molecular functions. “^∗^” indicates significantly enriched GO terms, of which the *P*-value < 0.05. **(B)** The top 20 functionally enriched KEGG analysis of DMGs.

We further functionally categorized the DMGs and analyzed their significant differences by using the GOseq R package (Young et al., 2010). These DMGs were assigned to one or more of three categories: biological process, cellular component, and molecular function base on the GO assignments, and they were significantly enriched in 25, 132, and 198 terms of three GO categories in AZD vs. DMSO, RAP vs. DMSO, and AZD + RAP vs. DMSO groups, respectively (corrected *P* < 0.05) (Supplementary Table S3). The top three significantly enriched GO terms were “cell periphery,” “plasma membrane,” and “catalytic activity” in AZD vs. DMSO group, “catalytic activity,” “nucleotide binding,” and “nucleoside phosphate binding” in RAP vs. DMSO group, and “nucleotide binding,” “nucleoside phosphate binding,” and “ribonucleoside binding” in AZD + RAP vs. DMSO group (Figure 6A and Supplementary Figure S3A), suggesting that these GO terms may play important roles in TOR-regulated genomic methylation. Furthermore, the largest number of functional GO term was “cell” under TOR inhibition, which distributed in the cellular component category, implying that TOR may participate in the regulation of cellular component GO terms.

To provide further insight into the pathways, we performed KEGG pathway analysis of the DMGs under TOR inhibition. The major metabolic pathways and signal transduction pathways of DMGs were identified by KEGG significant enrichment. The top two enriched KEGG pathways were “Carbon metabolism” and “Biosynthesis of amino acids” under TOR inhibition (Figure 6B and Supplementary Figure S3B). In addition, DMGs in “RNA transport,” “Ribosome biogenesis in eukaryotes,” and “beta-Alanine metabolism” pathways were also found under TOR inhibition.

### DMGs Involved in the Regulation of Cell Growth

Carbon metabolism and synthesis of proteins are important limiting factors for cell growth and proliferation (Webb and Satake, 2015; Saxton and Sabatini, 2017). Among these altered metabolic processes in KEGG pathways, the number of “Carbon metabolism” and “Biosynthesis of amino acids” pathways was the largest (Figure 6B), indicating that TOR controlled cell growth and proliferation by regulating the methylation level of the genes. We further analyzed the methylation levels of “Carbon metabolism” and “Biosynthesis of amino acids” pathways in RAP vs. DMSO group. A total of 21 and 17 DMGs had significant changed methylation levels in “Carbon metabolism” and “Biosynthesis of amino acids” pathways, respectively (methylation ratio > 1.5-fold) (Table 3). The genes encoding rate-limiting enzymes of carbon metabolism and biosynthesis of amino acids such as 6-phosphofructokinase (*PFK6*) and isocitrate dehydrogenase (*IDH5*) were hypermethylated, suggesting that TOR inhibition by RAP reduced the carbon metabolism levels in *Arabidopsis*. In addition, the methylation levels of genes in “Carbon metabolism” and “Biosynthesis of amino acids” pathways were also changed in AZD-treated samples (Supplementary Table S4). These results indicated that TOR regulated multiple metabolic processes by altering the methylation levels of related genes.

**TABLE 3:** Differentially methylated genes (DMGs) of carbon metabolism, biosynthesis of amino acids and ribosome in RAP vs. DMSO group.

**Gene id**	**Methylation ratio**	**Status**	**Regions**	**Annotation**
**Carbon metabolism**				
AT1G17745	0.0065	Hypo	Promoter	PGDH2| Allosteric substrate binding domain
AT3G52200	0.3913	Hypo	Exon/intron	LTA3| 2-oxoacid dehydrogenase acyltransferase
AT5G08300	0.5078	Hypo	Promoter	ATP-citrate lyase/succinyl-CoA ligase
AT1G04410	0.5323	Hypo	Promoter	MDH1| Lactate dehydrogenase/glycoside hydrolas
AT4G13890	0.5464	Hypo	Exon	SHM5| Pyridoxal phosphate-dependent transferase
AT5G23250	0.6175	Hypo	Exon/intron/utr3	ATP-citrate lyase/succinyl-CoA ligase
AT4G26970	0.6402	Hypo	Exon/intron	ACO3| Aconitase/3-isopropylmalate dehydratase
AT1G22020	0.6478	Hypo	Exon/intron	SHM6| Pyridoxal phosphate-dependent transferase
AT5G11670	0.6696	Hypo	Promoter	NADP-ME2| Malic enzyme, NAD-binding
AT1G79530	0.6699	Hypo	Promoter	GAPCP1| Glyceraldehyde 3-phosphate dehydrogenase
AT2G07732	1.5040	Hyper	Promoter	Ribulose bisphosphate carboxylase, large subunit
AT4G32840	1.5343	Hyper	Promoter	PFK6| Phosphofructokinase
AT2G36460	1.5464	Hyper	Exon	Fructose-bisphosphate aldolase
AT1G54220	1.5971	Hyper	Promoter	2-oxoacid dehydrogenase acyltransferase
AT1G36370	1.6290	Hyper	Exon	SHM7| Pyridoxal phosphate-dependent transferase
AT1G74030	1.6688	Hyper	Exon/intron/utr3	ENO1| Enolase
AT3G49360	1.9074	Hyper	Promoter	PGL2| 6-phosphogluconolactonase, DevB-type
AT5G03290	1.9497	Hyper	Exon	IDH5| Isocitrate dehydrogenase NAD-dependent
AT3G12780	1.9568	Hyper	Exon/intron	PGK1| Phosphoglycerate kinase
AT1G01090	2.0177	Hyper	Exon/intron	PDH-E1| Pyruvate dehydrogenase E1 component
AT1G17650	2.6505	Hyper	Exon/intron	GLYR2| 6-phosphogluconate dehydrogenase
**Biosynthesis of amino acids**				
AT5G11880	0.1681	Hypo	Exon/intron	LYSA2| Diaminopimelate decarboxylase, LysA
AT1G58080	0.2884	Hypo	Exon/intron	HISN1A| ATP phosphoribosyltransferase
AT3G22425	0.4460	Hypo	Promoter	HISN5A| Imidazoleglycerol-phosphate dehydratase
AT4G13890	0.5464	Hypo	Exon	SHM5| Pyridoxal phosphate-dependent transferase
AT4G37670	0.5988	Hypo	Exon/intron	NAGS2| Acyl-CoA *N*-acyltransferase
AT4G26970	0.6402	Hypo	Exon/intron	ACO3| Aconitase dehydratase large subunit
AT1G22020	0.6478	Hypo	Exon/intron	SHM6| Pyridoxal phosphate-dependent transferase
AT1G79530	0.6699	Hypo	Promoter	GAPCP1| Glyceraldehyde 3-phosphate dehydrogenase
AT4G32840	1.5343	Hyper	Promoter	PFK6| Phosphofructokinase
AT2G36460	1.5464	Hyper	Exon	Fructose-bisphosphate aldolase, class-I
AT1G36370	1.6290	Hyper	Exon	SHM7| Pyridoxal phosphate-dependent transferase
AT1G74030	1.6688	Hyper	Exon/intron/utr3	ENO1| Enolase
AT2G45440	1.6816	Hyper	Intron	DHDPS2| Dihydrodipicolinate synthase, DapA
AT4G01850	1.7869	Hyper	Exon	SAM2| S-adenosylmethionine synthetase
AT4G23590	1.9377	Hyper	Exon	Pyridoxal phosphate-dependent transferase
AT5G03290	1.9497	Hyper	Exon	IDH5| Isocitrate dehydrogenase NAD-dependent
AT3G12780	1.9568	Hyper	Exon/intron	PGK1| Phosphoglycerate kinase
**Ribosome**				
AT4G27490	0.2287	Hypo	Promoter	Ribosomal protein S5 domain 2-type fold
AT1G23280	0.2364	Hypo	Promoter	Mak16| Ribosomal L28e
AT1G52930	0.2828	Hypo	Exon/intron	BRX1| Ribosome biogenesis protein
AT5G59180	0.3100	Hypo	Exon/utr3	NRPB7| Ribosomal protein S1, RNA-binding domain
AT1G04170	0.3194	Hypo	Promoter	EIF2γ| Translation elongation factor EF1A gamma
AT1G32990	0.4472	Hypo	Promoter	RPL11| Ribosomal protein L11
AT1G07210	0.4729	Hypo	Exon/intron	Ribosomal protein S18
AT5G05470	0.4853	Hypo	Exon/intron	EIF2α| Translation initiation factor 2, alpha subunit
AT4G10450	0.5106	Hypo	Promoter	RPL9D| Ribosomal protein L6
AT1G07770	0.5567	Hypo	Exon/intron	RPS15AA| Ribosomal protein S8
AT3G06580	0.5818	Hypo	Exon/intron	GAL1| Ribosomal protein S5 domain 2-type
AT1G02830	0.6042	Hypo	Promoter	RPL22A| Ribosomal protein L22e
AT3G63490	0.6304	Hypo	Exon/intron/utr3	RPL1| Ribosomal protein L1
AT2G44860	0.6369	Hypo	Exon/utr3	Ribosomal protein L24e, conserved site
AT2G25210	0.6443	Hypo	Exon/intron/utr5/promoter	Ribosomal protein L39e
AT5G64650	0.6492	Hypo	Exon/intron	Ribosomal protein L17
AT1G41880	1.5163	Hyper	Exon/utr3	RPL35AB| Ribosomal protein L35Ae
AT1G24240	1.5289	Hyper	Promoter	Ribosomal protein L19
AT3G10950	1.5472	Hyper	Promoter	RPL37AB| Ribosomal protein L37ae
AT1G31355	1.5676	Hyper	Promoter	Translation protein SH3-like family protein
AT4G16030	1.5756	Hyper	Promoter	Ribosomal protein L19/L19e
AT5G16130	1.5889	Hyper	Promoter	RPS7C| Ribosomal protein S7e
AT3G49010	1.6060	Hyper	Promoter	RPL13B| Ribosomal protein L13e
AT1G13950	1.6211	Hyper	Promoter	ELF5A-1| Ribosomal protein L2 domain 2
AT5G02870	1.7028	Hyper	Promoter	RPL4D| 60S ribosomal protein L4, C-terminal domain
AT1G26630	1.7231	Hyper	Exon/intron	ELF5A-2| Ribosomal protein L2 domain 2|
AT5G53920	1.8060	Hyper	Promoter	Ribosomal protein L11 methyltransferase
AT2G45030	1.9131	Hyper	Exon/utr3	MEFG2| Ribosomal protein S5 domain 2-type fold
AT3G20260	1.9516	Hyper	Promoter	Ribosomal protein L34Ae
AT2G40205	1.9979	Hyper	Promoter	RPL41E| Ribosomal protein L41
AT4G34730	2.1114	Hyper	Intron	Ribosome-binding factor A
AT1G31355	2.1706	Hyper	Promoter	Translation protein SH3-like family protein
AT5G19720	2.2472	Hyper	Promoter	Ribosomal protein L25, beta-barrel domain
AT1G01220	2.4560	Hyper	Promoter	FKGP| Ribosomal protein S5 domain 2-type fold
AT4G29060	2.9800	Hyper	Exon/utr3	emb2726| Ribosomal protein S1
AT2G20060	2.9911	Hyper	Promoter	Ribosomal protein L4
AT3G53890	3.4514	Hyper	Exon/utr3	RPS21B| Ribosomal protein S21e
AT5G39785	6.5648	Hyper	Exon/intron	Ribosomal protein L34Ae

The ribosome, composed of ribosomal RNAs and ribosomal proteins, is responsible for the synthesis of proteins in prokaryotes and eukaryotes (Adam et al., 2011; Opron and Burton, 2018). TORC1 positively regulates multiple steps including ribosomal RNAs transcription, the synthesis of ribosomal proteins and other components in ribosome biogenesis (Iadevaia et al., 2014; Kos-Braun and Kos, 2017). We found that 38 DMGs associated with ribosome genes in RAP vs. DMSO group (Table 3). Besides, a large number of DMGs associated with ribosome were also found in AZD and AZD + RAP treated samples (Supplementary Tables S4, S5). Interestingly, “Ribosome biogenesis in eukaryotes” was the most enriched pathway in AZD + RAP vs. DMSO group, of which 31 DMGs were found in this pathway (Supplementary Figure S3B and Table S5). Additionally, we found that the methylation level of *TOR* was reduced whereas the transcription level of *TOR* was up-regulated under TOR inhibition (Supplementary Figure S1B), suggesting a feedback regulation of TOR inhibition in *Arabidopsis*. Collectively, these results and observations suggested that TOR plays a crucial role in plant growth and development through regulating multiple metabolic processes and protein synthesis.

### DMGs Involved in the Regulation of Plant Hormone Signal Transduction

Plant hormones play indispensable roles in mediating cellular metabolism, regulating plant growth and development, and resisting biotic and abiotic stresses (Rubio et al., 2009). Based on the WGBS data, DMGs associated with auxin, cytokinin (CK), brassinosteroid (BR), abscisic acid (ABA), ethylene (ET), and jasmonic acid (JA) were detected under TOR inhibition (Supplementary Table S6). Among these phytohormone signaling pathways, the top three largest number of DMGs were CK, BR, and ABA signaling pathways. Recent studies showed that TOR interacted with ABA signaling to balance plant growth and stress responses in plants (Wang et al., 2018). Based on our data, several ABA signaling pathway-related genes were significantly differentially methylated. In detail, the protein kinase *SnRK2* of the ABA signaling pathway was hypermethylated, whereas protein phosphatase *PP2CA* was hypomethylated. Besides, some important plant hormone-related genes were differentially methylated. For example, auxin responsive SAUR proteins were hypermethylated in the promoter region, and BR signaling protein kinases *BSK1* and *BSK2* were hypomethylated under TOR inhibition (Supplementary Table S6). The transcription levels of *ABI5*, *BSK2*, and *PP2CA* genes were up-regulated whereas methylation levels of these genes were decreased in the promoter regions under TOR inhibition (Supplementary Figure S2C and Supplementary Table S6). These results showed that TOR may act as a regulator to mediate plant hormone signals transduction in *Arabidopsis*.

### Association of DMGs With Gene mRNA Expression Level

**FIGURE 7:**
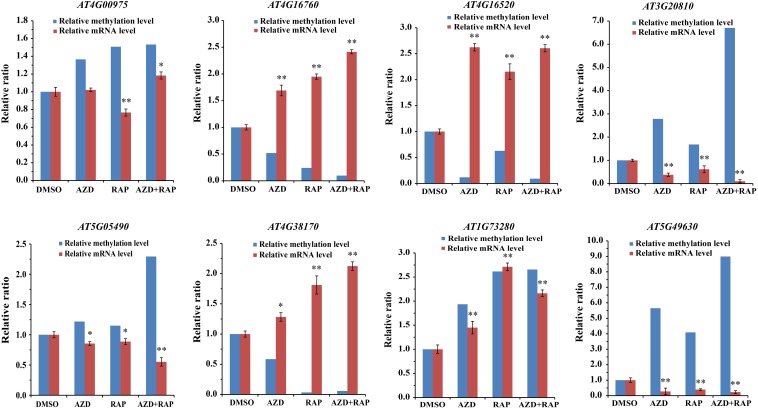
Relationship between DNA methylation and gene mRNA expression level in DMSO, AZD, RAP, and AZD + RAP treated BP12-2 samples. Error bars indicate means ± SD of three biological replicates. Asterisks denote Student’s *t*-test significant difference compared with DMSO (^∗^*P* < 0.05, ^∗∗^*P* < 0.01).

To dissect the relationship between DMGs and gene mRNA expression level, we examined the expression levels of related genes using qRT-PCR. Eight DMGs were randomly selected for the real-time PCR, of which three DMGs involved in stresses response and five DMGs involved in metabolism and cell growth. Consistent with the previous study (Zhang et al., 2006), gene mRNA expression level was decreased in the hypermethylated promoter region in this study (Figure 7). For example, *AT4G16520* (*ATG8F*) and *AT4G16760* (*ACX1*) induced by stresses were hypomethylated in the promoter region, while mRNA expression level was upregulated under TOR inhibition. *AT5G05490* (*SYN1*) and *AT5G49630* (*AAP6*) that involved in cell growth were hypermethylated whereas mRNA expression level was downregulated. Besides, some genes hypermethylated in transcribed regions were highly expressed whereas other genes were low expressed (Figure 7 and Supplementary Table S7), demonstrating methylation in transcribed regions both positive and negative relationships to gene expression (Zhang et al., 2006; Lou et al., 2014).

## Discussion

In this study, we analyzed the functions of TOR in the regulation of DNA methylation using WGBS. We found that inhibition of TOR reduced whole-genome methylation levels whereas the methylation level of CHH site in the promoter region was increased. CHH methylation is maintained by DRM1 or DRM2 in plants. Through RNA-directed DNA methylation (RdDM) pathway, DRM2 maintains CHH methylation at RdDM target regions (Zhang et al., 2018). The transcription level of *MET1* gene was down-regulated whereas *DRM1* and *DRM2* genes were up-regulated under TOR inhibition. Furthermore, the transcription level of DNA demethylation genes were significantly up-regulated in TOR-inhibited seedlings. These results explained that inhibition of TOR results in lower genome-wide methylation levels but increases methylation level of CHH site in the promoter region. Besides, CHROMOMETHYLASE 2 (CMT2) is also involved in maintaining CHH methylation in plants (Zemach et al., 2013; Stroud et al., 2014). Methylation level of *CMT2* was decreased in AZD + RAP treated sample, implying that TOR inhibition may activate DRM2 or CMT2 to maintain CHH methylation level. Interestingly, our study showed that TOR regulated genome DNA methylation to control plant growth in *Arabidopsis*, while curcumin induced the promoter hypermethylation of *mTOR* gene in myeloma cells (Chen et al., 2019), suggesting that TOR had a feedback regulation mechanism in the process of regulating DNA methylation. The detailed regulatory mechanisms of TOR and DNA methyltransferases still need further study in the future.

In addition to reduced genome-wide methylation, we also identified 1296, 4015, and 4520 DMGs in AZD vs. DMSO, RAP vs. DMSO, and AZD + RAP vs. DMSO groups, respectively. The difference of DMGs between AZD and RAP may be caused by off-target effects in *ScFKBP12*-overexpressed *Arabidopsis*. Previous studies suggested that the expression of *FKBP12* in *Arabidopsis* might have unexpected molecular phenotypes unrelated to TOR signaling pathway due to its peptidyl-prolyl isomerase activity (Gerard et al., 2011; Alavilli et al., 2018). Changes of non-TOR-kinase specific in intracellular metabolism caused by RAP off-targets in *ScFKBP12*-overexpressed *Arabidopsis* still need further study.

TOR signaling is indispensible for growth and development from embryogenesis to senescence by modulating translation, autophagy, metabolism, and cell cycle in plants (Ren et al., 2012; Xiong and Sheen, 2014; Shi et al., 2018). In our study, many genes of cellular metabolic processes and signal pathways were differentially methylated under TOR inhibition, especially carbon metabolism and biosynthesis of amino acids. Furthermore, DMGs associated with ribosome and ribosome biogenesis were detected. It is well known that TOR controls protein synthesis at multiple levels from transcription, ribosome biogenesis to protein translation in various eukaryotes (De Virgilio and Loewith, 2006; Xiong et al., 2013; Yang et al., 2013; Xiong and Sheen, 2014; Dong et al., 2015; Li et al., 2019). Our results indicated that TOR involved in the regulation of ribosome and ribosome biogenesis by changing the methylation levels of related genes, which is responsible for protein synthesis and plant growth.

Plant hormones play essential roles in plant growth, development and reproduction (Durbak et al., 2012). Previous studies demonstrate that TOR is indispensable for auxin signaling transduction, and auxin activates TOR to promote translation reinitiation in *Arabidopsis* (Deng et al., 2016; Schepetilnikov et al., 2017). Moreover, TOR signaling also promotes accumulation of BZR1 protein to promote plant growth in *Arabidopsis* (Zhang et al., 2016). Nevertheless, TOR signal and ABA or JA signal are antagonism to balance plant growth and stress response (Song et al., 2017; Wang et al., 2018). Based on the WGBS data, we found some DMGs in plant hormone signal transduction including auxin, BR and ABA signals. The differential methylation of these genes may result in changes in gene expression level, providing a new insight of the involvement of TOR in phytohormone signaling.

In summary, DNA methylation inhibitor azacitidine and TOR inhibitors synergistically inhibited the growth of *Arabidopsis* seedlings, implying that TOR played a role in DNA methylation. We therefore further systematically investigated changes in genome DNA methylation levels under TOR inhibition by high-throughput bisulfite sequencing, and we obtained a large number of differentially methylated regions and genes. Based on the whole-genome DNA methylation data, hypomethylation level of whole-genome DNA was observed in AZD, RAP, and AZD + RAP treated *Arabidopsis*. KEGG pathway enrichment showed that DMGs were involved in many metabolic pathways, such as carbon metabolism and biosynthesis of amino acids. Additionally, we also found that some plant hormone signal transduction-related genes displayed significant differences in methylation level under TOR inhibition. In conclusion, the above studies revealed the genome methylation pattern under TOR inhibition, providing important clues for further analysis of the functions of TOR in DNA methylation.

## Data Availability Statement

The datasets generated for this study can be found in the NCBI Sequence Read Archive (SRA) accession: PRJNA606264.

## Author Contributions

MR, TZ, and LL designed the experiments. TZ and LL performed the experiments. LL, LF, and HM analyzed the data. MR, TZ, and LL wrote the manuscript.

## Conflict of Interest

The authors declare that the research was conducted in the absence of any commercial or financial relationships that could be construed as a potential conflict of interest.

## Footnotes

1 http://www.genome.jp/kegg/
