# Supplementary material for: Target of Rapamycin Regulates Genome Methylation Reprogramming to Control Plant Growth in Arabidopsis
Source: Front Genet. 2020 Mar 3;11:186. doi: 10.3389/fgene.2020.00186 (PMC7062917; doi:10.3389/fgene.2020.00186)
Supplement: Supplementary file 1 [file Presentation_1.pdf]

Target of rapamycin regulates genome methylation reprogramming to control plant growth in *Arabidopsis*

Tingting Zhu[1,2+], Linxuan Li[1+], Li Feng[1,3], Huijuan Mo[3], and Maozhi Ren[1,3*]

1 Institute of Urban Agriculture, Chinese Academy of Agricultural Sciences, Chengdu, 610213, China
2 School of Life Sciences, Chongqing University, Chongqing, 400045, China
3 Zhengzhou Research Base，State Key Laboratory of Cotton Biology, Zhengzhou University, Zhengzhou, 450000, China

Supplementary Figures:

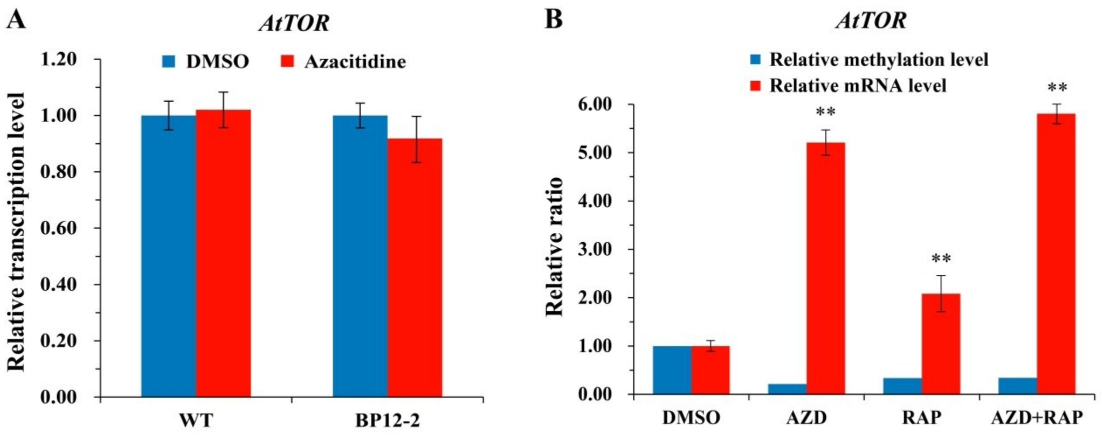

Supplementary Figure 1 | The transcription level of *AtTOR* in azacitidine- and TOR inhibitors-treated seedlings.

(A) The relative transcription level of *AtTOR* in 10 μM azacitidine-treated WT and BP12-2 seedlings.

(B) The relative methylation level and mRNA level of *AtTOR* in TOR inhibitors-treated BP12-2 seedlings. Error bars indicate means ±SD of three biological replicates. Asterisks denote Student's *t*-test significant difference compared with DMSO (**P < 0.01).

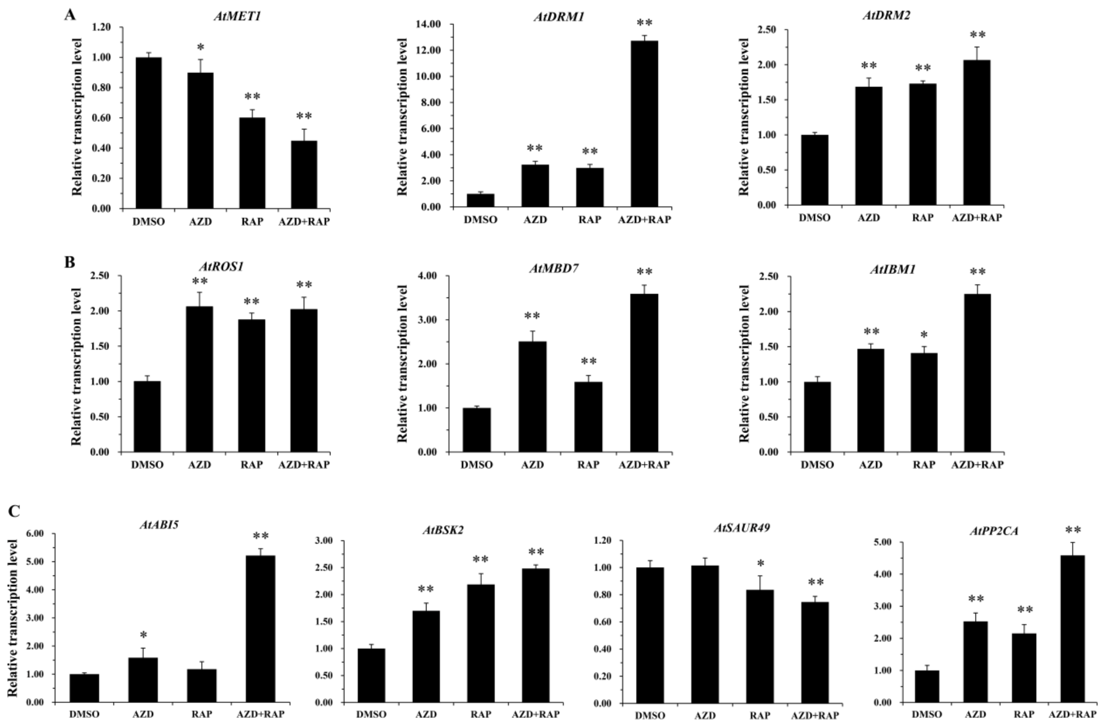

Supplementary Figure 2 | The transcription levels of methyltransferase, demethylase and plant hormone related genes under TOR inhibition.

(A) and **(B)** The transcription levels of methyltransferase and demethylase genes in DMSO, AZD, RAP and AZD+RAP treated BP12-2 seedlings.

(C) The transcription levels of plant hormone related genes in DMSO, AZD, RAP and AZD+RAP treated BP12-2 seedlings. Error bars indicate means ±SD of three biological replicates. Asterisks denote Student's *t*-test significant difference compared with DMSO (*P < 0.05; **P < 0.01).

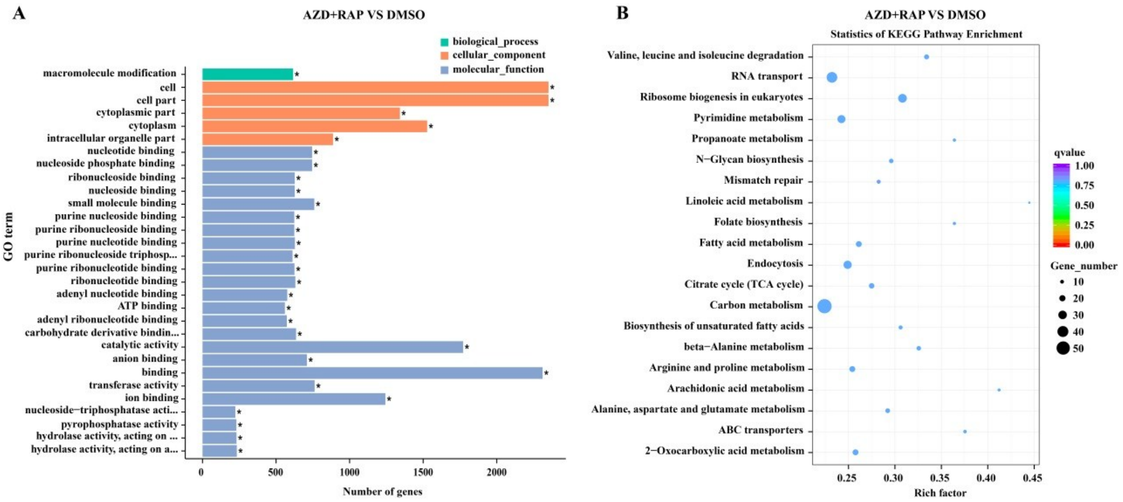

Supplementary Figure 3 | GO and KEGG pathway enrichment analysis of DMGs in AZD+RAP vs DMSO group.

(A) The top 30 most enriched GO terms analysis of DMGs in AZD+RAP vs DMSO

group. “*” indicates significantly enriched GO terms, of which the *P*-value < 0.05.
(B) The top 20 functionally enriched KEGG analysis of DMGs in AZD+RAP vs DMSO group.
