# Supplementary material for: Target of Rapamycin Regulates Genome Methylation Reprogramming to Control Plant Growth in Arabidopsis
Source: Front Genet. 2020 Mar 3;11:186. doi: 10.3389/fgene.2020.00186 (PMC7062917; doi:10.3389/fgene.2020.00186)
Supplement: Supplementary file 2 [file Table_1.DOCX]

Supplementary Table 1. Primers for qRT-PCR in this study

| Gene ID | Sequence (5’ to 3’) | Length |
| --- | --- | --- |
| RT AT4G16520 F | 5’-AAAGTACCTAGTCCCGGCTGAT-3’ | 22 |
| RT AT4G16520 R | 5’-AGGAAGAACATTGTCCACGAAT-3’ | 22 |
| RT AT4G16760 F | 5’-CATAGGTGGCAAAGGAGTGAAG-3’ | 22 |
| RT AT4G16760 R | 5’-TTGGGAGTGATGCAGTTAGTGG-3’ | 22 |
| RT AT4G00975 F | 5’-ACAGGCCCTAAACCCTTGTTG-3’ | 21 |
| RT AT4G00975 R | 5’-GAGAACCGAGCCGAGTAACCA-3’ | 21 |
| RT AT3G20810 F | 5’-CTGCGTGATTATTATCTGCC-3’ | 20 |
| RT AT3G20810 R | 5’-AGTGATTCCACTTGGTCCTG-3’ | 20 |
| RT AT5G05490 F | 5’-AACCACCATCAAACAGACCAT-3’ | 21 |
| RT AT5G05490 R | 5’-ACTGAAGCACCTTTCCCAGTA-3’ | 21 |
| RT AT4G38170 F | 5’-GGAGTTTGTTGAGACCCTTGC-3’ | 21 |
| RT AT4G38170 R | 5’-CTCCGAACTTAGCGACACGAT-3’ | 21 |
| RT AT1G73280 F | 5’-AAGTGGCTAGGCAAGCATCAAG-3’ | 22 |
| RT AT1G73280 R | 5’-GCCGGAACAACCATACCAGAGT-3’ | 22 |
| RT AT5G49630 F | 5’-CGGCAAACGCAACTACACCT-3’ | 20 |
| RT AT5G49630 R  RT AtACTIN2 F | 5’-CCCGTATTGAGCCAATCCAC-3’  5’-GCACTTGCACCAAGCAGCAT-3’ | 20  20 |
| RT AtACTIN2 R  RT AtTOR F  RT AtTOR R  RT AtMET1 F  RT AtMET1 R  RT AtDRM1 F  RT AtDRM1 R  RT AtDRM2 F  RT AtDRM2 R  RT AtROS1 F  RT AtROS1 R  RT AtMBD7 F  RT AtMBD7 R  RT AtIBM1 F  RT AtIBM1 R  RT AtBSK2 F  RT AtBSK2 R  RT AtSAUR49 F  RT AtSAUR49 R  RT AtPP2CA F  RT AtPP2CA R  RT AtABI5 F  RT AtABI5 R | 5’-CCTTTCAGGTGGTGCAACGAC-3’  5’-TTGGGATGCGTGCCTTACTT-3’  5’-CAAGAGTCCCAAGACCCCAC-3’  5’-TGGGTAAGGTTGGAATGTGCT-3’  5’-CTGCCTGTGCTTGTGATTTATGT-3’  5’-GGAATTTGGTTTGGGTAGGT-3’  5’-TGTAGCGATCAGTTGTGCTG-3’  5’-CGAGCCGATTCGTCTACCA-3’  5’-GTGAGGGCGACATTCTCATAG-3’  5’-ATGTCGTTGGCTTCCCAGTT-3’  5’-TGTGATTGTGATCGGGTGGG-3’  5’-AGCCAGGAACAGGGAATAAA-3’  5’-CTCCGAATGCACCATAGAAA-3’  5’-CTGAAAGAAGAATATGGCATTGA-3’  5’-TAACTTGATGAGGGCAACCTAC-3’  5’-GGACCAAGTGGATCAGGAAAT-3’  5’-AATGCAGCTAGGACTAAACCC-3’  5’-GAATCGTCGGAGATACGTTGTC-3’  5’-ATACTCTTCCTCAGCCTCAGCT-3’  5’-TATTGGGATGGAGCTAGGGTT-3’  5’-TCACTCGCCAAGATCAAACAC-3’  5’-GAGAATGCGCAGCTAAAACA-3’  5’-GTGGACAACTCGGGTTCCTC-3’ | 21  20  20  21  23  20  20  19  21  20  20  20  20  23  22  21  21  22  22  21  21  20  20 |
